# Recovery of muscle function after deep neuromuscular block by means of diaphragm ultrasonography and adductor of pollicis acceleromyography with comparison of neostigmine vs. sugammadex as reversal drugs: study protocol for a randomized controlled trial

**DOI:** 10.1186/s13063-018-2525-7

**Published:** 2018-02-21

**Authors:** Iacopo Cappellini, Fabio Picciafuochi, Daniele Ostento, Ginevra Danti, Angelo Raffaele De Gaudio, Chiara Adembri

**Affiliations:** Department of Health Sciences, Section of Anesthesiology and Critical Care, University of Florence, Largo Brambilla 3, 50134 Florence, Italy

**Keywords:** Diaphragm ultrasonography, Sugammadex, Postoperative residual, Curarization, Neuromuscular monitoring

## Abstract

**Background:**

The extensive use of neuromuscular blocking agents (NMBAs) during surgical procedures still leads to potential residual paralyzing effects in the postoperative period. Indeed, neuromuscular monitoring in an intra-operative setting is strongly advocated. Acetylcholinesterase inhibitors can reverse muscle block, but their short half-life may lead to residual curarization in the ward, especially when intermediate or long-acting NMBAs have been administered. Sugammadex is the first selective reversal drug for steroidal NMBAs; it has been shown to give full and rapid recovery of muscle strength, thus minimizing the occurrence of residual curarization. Acceleromyography of the adductor pollicis is the gold standard for detecting residual curarization, but it cannot be carried out on conscious patients. Ultrasonography of diaphragm thickness may reveal residual effects of NMBAs in conscious patients.

**Methods/design:**

This prospective, double-blind, single-center randomized controlled study will enroll patients (of American Society of Anesthesiologists physical status I–II, aged 18–80 years) who will be scheduled to undergo deep neuromuscular block with rocuronium for ear, nose, or throat surgery. The study’s primary objective will be to compare the effects of neostigmine and sugammadex on postoperative residual curarization using two different tools: diaphragm ultrasonography and acceleromyography of the adductor pollicis. Patients will be extubated when the train-of-four ratio is > 0.9. Diaphragm ultrasonography will be used to evaluate the thickening fraction, which is the difference between the end expiratory thickness and the end inspiratory thickness, normalized to the end expiratory thickness. Ultrasonography will be performed before the initiation of general anesthesia, before extubation, and 10 and 30 min after discharging patients from the operating room. The secondary objective will be to compare the incidence of postoperative complications due to residual neuromuscular block between patients who receive neostigmine and those who receive sugammadex.

**Discussion:**

Postoperative residual curarization is a topic of paramount importance, because its occurrence can cause complications and increase the length of stay in hospital and the related costs. Diaphragm ultrasound assessment may become a bedside integrative tool in the neuromuscular monitoring field to detect concealed residual curarization in surgical patients who have received paralyzing agents.

**Trial registration:**

EudraCT, 2013-004787-62. Registered on 18 June 2014, as “Evaluation of muscle function recovery after deep neuromuscular blockade by acceleromyography of the adductor pollicis or diaphragmatic echography: comparison between sugammadex and neostigmine.”

ClinicalTrials.gov, NCT02698969. Registered on 15 February 2016, as “Recovery of Muscle Function After Deep Neuromuscular Block by Means of Diaphragm Ultrasonography and Adductor Pollicis Acceleromyography: Comparison of Neostigmine vs. Sugammadex as Reversal Drugs.”

**Electronic supplementary material:**

The online version of this article (10.1186/s13063-018-2525-7) contains supplementary material, which is available to authorized users.

## Background

Non-depolarizing neuromuscular blocking agents (NMBAs) are extensively used by anesthesiologists to maintain deep neuromuscular block (dNMB) during surgical operations. To avoid the postoperative residual effects of NMBAs, muscle relaxants should be fully catabolized to inactive metabolites prior to extubation. Nevertheless, when a patient is waking from general anesthesia, it is possible that some of the administered paralyzing agent is not completely transformed to its inactive form at the level of neuromuscular junctions, causing residual effects that can be difficult to clinically diagnose without adequate neuromuscular monitoring [1].

The use of intra-operative neuromuscular monitoring when NMBAs are administered has been encouraged in order to decrease postoperative residual curarization (PORC) [2]. Acceleromyography, the most commonly used method of quantitative monitoring, appraises muscle acceleration responding to nerve stimulation by train-of-four (TOF) and post-tetanic count (PTC) methods [3]. For many years, a TOF ratio not greater than 0.9 between the amplitude of the last stimulation and that of the first one was used to define PORC. Despite the fact that these types of monitoring are strongly recommended, they are not regularly performed in the operating room scenario [4, 5]. Furthermore, the TOF test is unpleasant for patients when they are conscious.

The incidence of PORC ranges from 9 to 56.5% when no reversal drug is administered [6]. For many years, reversing the NMBA effect has been carried out using an acetylcholinesterase inhibitor (AChEI) such as neostigmine. These inhibitors, which increase acetylcholine levels in the neuromuscular junction, antagonize the paralyzing agent but do not hasten its metabolism. Therefore, as a result of the unpredictable metabolism of blocking drugs, a residual curarization may occur when the AChEI effect has elapsed [7]. Indeed, an observational study showed that, 20 min after administration of neostigmine, 18% of patients had a TOF ratio < 0.9 [8]. Moreover, as shown in an animal study, when neostigmine is administered in the setting of full recovery from a muscle relaxant, the drug can cause weakness of the diaphragm and genioglossus muscle even if this effect is not seen when residual curarization is still present [9].

Sugammadex is the first selective reversal agent for steroidal NMBAs. It has been shown to give full and rapid recovery of muscle strength, thus minimizing the occurrence of PORC [8, 10].

The diaphragm, the major respiratory muscle in humans, is a great septum between the thoracic and abdominal cavities. The movement of the diaphragm accounts for 60–70% of the total tidal volume of respiration. Failure of diaphragmatic function is believed to play a central role in the pathophysiology of the clinical syndrome known as “pump respiratory failure” [11–13]. Although a TOF ratio > 0.9 in the adductor pollicis rules out residual curarization, the diaphragm is often not evaluated in the operating room. The diaphragm is the most highly resistant muscle to NMBAs, as well as the first to recover [14], but the occurrence of its dysfunction has been implicated in postoperative respiratory failure, especially when mechanical ventilation is prolonged [15, 16]. Therefore, studying diaphragmatic function in a perioperative context is extremely important.

Since 1985, ultrasonography has been used to evaluate diaphragm function by measuring thickness variations in the apposition zone, which reflect the extent of contraction of the muscle [17]. Vivier et al. recently demonstrated that the thickening fraction (TF), namely the difference between the thickness at the end of inspiration (TEI) and that at the end of expiration (TEE), normalized for TEE (TEI – TEE/TEE), is directly related to respiratory workload, and they suggested that TF could be used as an index to select those patients ready to be weaned from non-invasive ventilation [18]. These data suggest that ultrasound TF could also be used in different scenarios, and our purpose is to use ultrasound TF to assess diaphragm recovery after dNMB since it is more comfortable for conscious patients than acceleromyography.

We therefore hypothesize that the incidence of postoperative diaphragmatic dysfunction, assessed using ultrasonography and, from our unpublished observations, defined by a fractional shortening of the diaphragm < 40%, is lower in patients who receive sugammadex than in those who receive neostigmine.

## Methods/design

### Study design and eligibility

This study is a prospective, double-blind, randomized controlled trial involving 60 patients with American Society of Anesthesiologists (ASA) physical status I–II and aged between 18 and 80 years, who will undergo dNMB (standard care in our clinic) with rocuronium during ear, nose, or throat surgery in a university hospital. The Institutional Review Board of the Tuscany Region has approved the protocol with registration number CE SPE 13.068. Exclusion criteria are a history of hepatic or renal disease, chronic or acute alcoholism, allergy or hypersensitivity to sugammadex or neostigmine, current medication with effects on the central nervous system, a history of neurologic disease, diaphragmatic palsy, women who are pregnant or nursing, and arrhythmias.

### Randomization

Written informed consent will be obtained during the preoperative evaluation by an anesthesiologist working in the anesthesia unit of the hospital. Afterwards, each patient will be randomly allocated to either the sugammadex (SUG) group or the neostigmine (NEO) group. Randomization will be performed using a table created on www.randomization.com. The allocation plan will be carried out using a variable block randomization method 1:1 to distribute the patients equally to each group. For allocation concealment, table assignment to one group or the other will be managed by a pharmacist with limited involvement in the study; this person will also perform the allocation and prepare the drugs.

### Intervention plan

In order to standardize the anesthetic technique, no premedication will be administered. All patients will undergo neuromuscular monitoring with ulnar nerve stimulation using the TOF-Watch (Organon, Oss, Netherlands). The device will be calibrated preoperatively, and the parameters will be set using standard TOF methodology after administration of a hypnotic drug, prior to muscle relaxation. General anesthesia will be induced by intravenous injection of fentanyl (2 μg/kg body weight), propofol (2 mg/kg), and rocuronium (0.6 mg/kg). Tracheal intubation will be performed after the patient fails to register signals using TOF. To maintain dNMB, rocuronium (0.15 mg/kg) will be re-administered when the PTC elicits more than five twitches. Sevoflurane will be supplied at an age-adjusted end-tidal concentration of 1.0 minimum alveolar concentration (MAC) in an air/oxygen mixture. Fentanyl will be titrated with a bolus of 0.5 μg/kg every 30 min, to keep an adequate level of analgesia.

Prior to induction of anesthesia, the baseline TF will be evaluated by one operator skilled in ultrasonography using an Esaote MyLab ultrasound instrument (Esaote, Genoa, Italy). Patients will be placed on the bed in a semi-recumbent (45°) position, assessed by a goniometer. The operator will use a 10–12 MHz high-frequency linear probe to identify the diaphragm in the midaxillary line in the apposition zone between the lung and liver on the right and between the lung and spleen on the left, in the intercostal spaces between the ninth, tenth, and eleventh ribs, 0.5–2 cm above the costophrenic sinus. The TF will be calculated at tidal breathing, recorded in time-motion mode. The muscle will be located using the hyperechoic pleural and peritoneal layers. Three assessments will be performed in consecutive breaths and averaged [18].

**Fig. 1:**
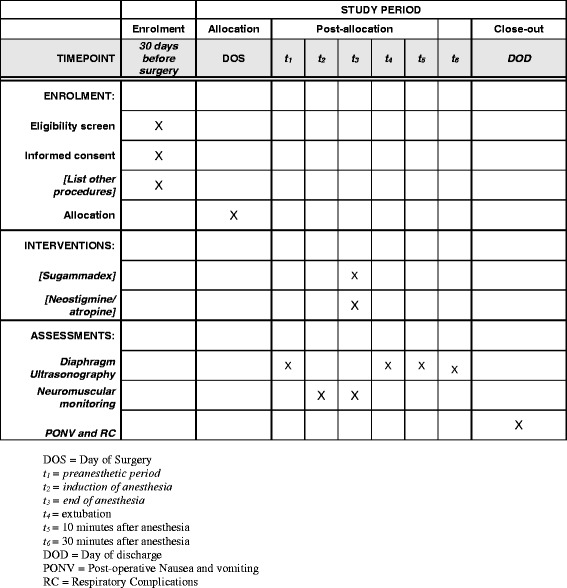
Study timeline

**Fig. 2:**
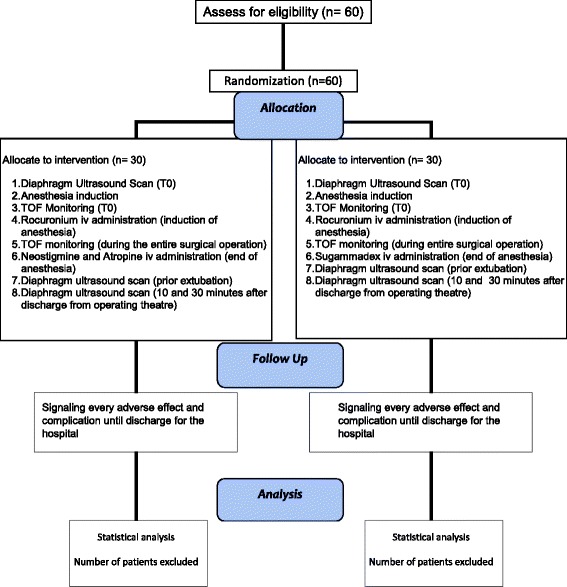
Study flow diagram

At the end of the operation and when TOF neuromuscular monitoring shows a minimum of two twitches, patients will receive the reversal drug according to the group to which they have been randomized. Patients in the NEO group will receive 50 μg/kg neostigmine and 15 μg/kg atropine, while those in the SUG group will receive 2 mg/kg sugammadex [19]. The drugs will be prepared for intravenous injection in identical volumes and in indistinguishable syringes so that the anesthesiologist will be blinded to the treatments the patients receive. Extubation will be performed when all the following criteria are met: (1) the patient is awake and can execute simple commands; (2) the patient’s respiratory pattern is regular with a tidal volume of 6–7 mL/kg referred to ideal body weight; (3) the TOF ratio is > 0.9. Immediately prior to extubation, bilateral diaphragm ultrasonography will be performed to assess muscle recovery in spontaneously breathing patients; these measurements will be compared to the baseline muscle assessment. Two additional diaphragm ultrasound scans will be performed 10 and 30 min after discharge from the operating theater under the same conditions as described above, but no further TOF monitoring will be carried out. Follow-up will be performed to document adverse events and complications until discharge from the hospital. The physician who performs the ultrasound scan will be different from the one who administers the reversal drug and will be blinded to the treatment that patients receive (Figs. 1 and 2). The Standard Protocol Items: Recommendations for Interventional Trials (SPIRIT) checklist is provided as Additional file 1.

In the case of unexpected events, such as a change in drug dose or a participant’s request to withdraw from the study, the protocol will be stopped and this will be recorded on the case report form (CRF). No further tests will be performed except those necessary to finalize the protocol. Should patients need medical care that interferes with the correct conduct of the study, they will be excluded from the study.

The primary endpoint of the study will be a 30% relative reduction in the incidence of residual curarization in patients who receive sugammadex compared with neostigmine 30 min after drug administration. The primary endpoint will be assessed at 30 min because the effects of neostigmine start to fade after this time has elapsed [20]. Residual curarization will be determined by the percentage of 30 min TF compared to baseline TF. It is not known exactly what percentage TF indicates residual curarization, but from our unpublished observations, values of 40% or less could indicate diaphragmatic dysfunction. However, we do not know what percentage of patients have a TF < 40% when neostigmine is administered.

Two secondary endpoints will be assessed. One secondary endpoint will be a 10% relative reduction in respiratory complications related to residual curarization obtained with sugammadex compared with neostigmine. Respiratory complications taken into consideration will be new cough and sputum production, abnormal breath sounds not present at baseline, temperature higher than 38 °C, chest radiography documentation of atelectasis or new infiltrates, and physician documentation of atelectasis or pneumonia [21]. The other secondary endpoint will be a 30% relative decrease in postoperative nausea or vomiting in patients who receive sugammadex compared with those who receive neostigmine.

Data monitoring will be performed by an anesthetist not involved in the study. Data will be collected on paper CRFs. All personal information will be registered in an environment limited to medical personnel to maintain absolute confidentiality. Data entry will be performed at one central site that maintains the overall database and will carry out the data analysis. All the compiled CRFs will be archived in a locker to which only clinicians involved in the study have access. In order to eliminate possible data entry errors, individual data will be compared to a range of plausible values. After data entry, automated checks, which have been defined a priori, will be performed to search for internal inconsistencies, range errors, or missing data. For each atypical, out-of-range, or missing datum, a query will be automatically sent to the investigator. Once all the queries are solved, the database will be locked and used for statistical analysis. All the individual participant data collected during the trial, after de-identification, will be available. The study protocol, statistical analysis plan, and analytic code will be accessed beginning 3 months and ending 5 years following article publication by researchers who will provide a methodologically sound proposal to achieve aims in the approved proposal. Each request must be sent to the corresponding author.

### Statistical analyses and sample size calculation

The statistical analysis will be performed by an independent statistician using SAS 9.3 (SAS Institute, Inc., Cary, NC, USA). For the primary endpoint, the effect of a drug on ΔTF will be estimated using generalized estimating equations (GEEs) and a multiple linear regression model adjusting for time of the measurement and baseline TF of the right side. This hemidiaphragm has been chosen because it is more easily identified for the presence of the liver. For the secondary endpoint, the association between a drug and its collateral effect will be evaluated using a logistic regression model. *P* values lower than 0.05 have been considered statistically significant.

Finally, descriptive statistics of all variables describing the characteristics of the patients enrolled in the study and those excluded from the study will be analyzed. Continuous variables will be expressed as mean (± standard deviation, (SD)) and median (ranging from 25th to 75th percentiles). Percentages will be calculated for dichotomous data. For categorical variables, frequency counts and percentages will be calculated.

Since this is the first clinical trial that proposes, as its primary endpoint, a relative reduction of 30% in the incidence of residual curarization, the necessary sample size has been calculated using the statistical software Epi Info (version 7). This analysis shows that at least 30 patients per group will be necessary, considering that in 5% of subjects TF is not valuable [22, 23], and expecting 23–25% of residual curarization with neostigmine 30 min after the extubation vs. 2–4% after the administration of sugammadex (with a 95% confidence interval (CI) and a power of 80%, and assuming equal variance between the two groups). For the secondary outcome, the number needed to treat has been calculated with a CI of 95%.

## Discussion

Diaphragm ultrasonography has been used for 25 years to evaluate diaphragmatic dysfunction in many clinical scenarios [18, 24–26]. Ultrasound assessment of the diaphragm is not feasible if the operator is not adequately trained, but when skilled operators are available, this tool enables bedside evaluation of the major respiratory muscle. We have observed (unpublished observations) that the reproducibility and repeatability of diaphragm ultrasonography are moderate when the test is performed by three different operators with different levels of experience with sonography. However, one potential limitation of the present protocol could be that previous studies have demonstrated that repeatability ranges from 13 to 19% [22].

This study will be the first to assess if TF measurements in the operating room enable physicians to diagnose and eventually treat residual curarization after dNMB. The rationale for comparing sugammadex to neostigmine is that the latter has a pharmacokinetic profile that cannot avoid residual curarization, especially when an intermediate or long-lasting muscle relaxant is administered during general anesthesia, while the former avoids residual curarization because it binds stably with steroid NMBA molecules by means of van der Waals and hydrophobic interactions [27]. Therefore, the present study will be the first that aims to detect diaphragmatic dysfunction using ultrasonography for the purpose of assessing PORC after deep neuromuscular blockade with an aminosteroid muscle relaxant drug.

### Trial status

Currently, patient recruitment is completed and all data have been collected. Data analysis will begin shortly.

## Additional file

Additional file 1: SPIRIT 2013 checklist: recommended items to address in a clinical trial protocol and related documents. (DOC 122 kb)

## Abbreviations

AChEI: Acetylcholinesterase inhibitor
ASA: American Society of Anesthesiologists
CI: Confidence interval
CRF: Case report form
dNMB: Deep neuromuscular block
MAC: Minimum alveolar concentration
NEO: Neostigmine
NMBA: Neuromuscular blocking agent
PORC: Postoperative residual curarization
PTC: post-tetanic count
SUG: Sugammadex
TEE: Thickness at the end of expiration
TEI: Thickness at the end of inspiration
TF: Thickening fraction
TOF: Train of four
